# Rapid closure technique in suboccipital decompression

**DOI:** 10.1007/s00068-021-01779-w

**Published:** 2021-09-25

**Authors:** Martin Vychopen, Alexis Hadjiathanasiou, Simon Brandecker, Valeri Borger, Patrick Schuss, Hartmut Vatter, Erdem Güresir

**Affiliations:** Department of Neurosurgery, University Hospital Bonn, Venusberg-Campus 1, 53127 Bonn, Germany

**Keywords:** Suboccipital, Decompression, Rapid closure

## Abstract

**Objective:**

Suboccipital decompression has been established as standard therapeutic procedure for raised intracranial pressure caused by mass-effect associated pathologies in posterior fossa. Several different surgical techniques of dural closure have been postulated to achieve safe decompression. The aim of this study was to examine the differences between fibrin sealant patch (FSP) and dural reconstruction (DR) in suboccipital decompression for acute mass-effect lesions.

**Methods:**

We retrospectively analyzed our institutional data of patients who underwent suboccipital decompression due to spontaneous intracerebellar hemorrhage, cerebellar infarction and acute traumatic subdural hematoma between 2010 and 2019. Two different dural reconstruction techniques were performed according to the attending neurosurgeon: (1) fibrin sealant patch (FSP), and (2) dural reconstruction (DR) including the use of dural patch. Complications, operative time, functional outcome and the necessity of a ventriculoperitoneal shunt (VP Shunt) were assessed and further analyzed.

**Results:**

Overall, 87 patients were treated at the authors’ institution (44 in FSP group, 43 in DR group). Glasgow coma scale on admission and preoperative coagulation state did not differ between the groups. Postoperatively, we found no difference in cerebrospinal fluid leakage or chronic hydrocephalus between the groups (*p* = 0.47). Revision rates were 2.27% (1/44 patients) in the FSP group, compared to 16.27% (7/43) in the DR group (*p* < 0.023). Operative time was significantly shorter in the FSP group (90.3 ± 31.0 min vs. 199.0 ± 48.8 min, *p* < 0.0001).

**Conclusion:**

Rapid closure technique in suboccipital decompression is feasible and safe. Operative time is hereby reduced, without increasing complication rates.

## Introduction

Suboccipital decompressive craniectomy (SDC) is widely used for patients suffering from raised intracranial pressure caused by cerebellar infarction [1, 2]. In a retrospective analysis of patients suffering from spontaneous cerebellar hemorrhage, SDC proved to be the most beneficial, compared to craniotomy and evacuation of haematoma, external ventricular drainage alone, or conservative treatment [3]. Similar results have also been published for patients with cerebellar infarction [13].

Various surgical techniques for watertight dural reconstruction were introduced in the literature [4–7]. However, the use of dural grafts is a time consuming procedure, which might cause additional complications [8]. For supratentorial craniectomy, a randomized trial showed rapid-closure technique to be safe procedure reducing operative time and costs of the operation without significantly higher incidence of surgical complications [9]. The necessity of dural graft in SDC is still underinvestigated. The aim of our study was to analyze and compare the use of fibrin sealant patch with surgical reconstruction using dural grafts.

## Methods

**Fig. 1:**
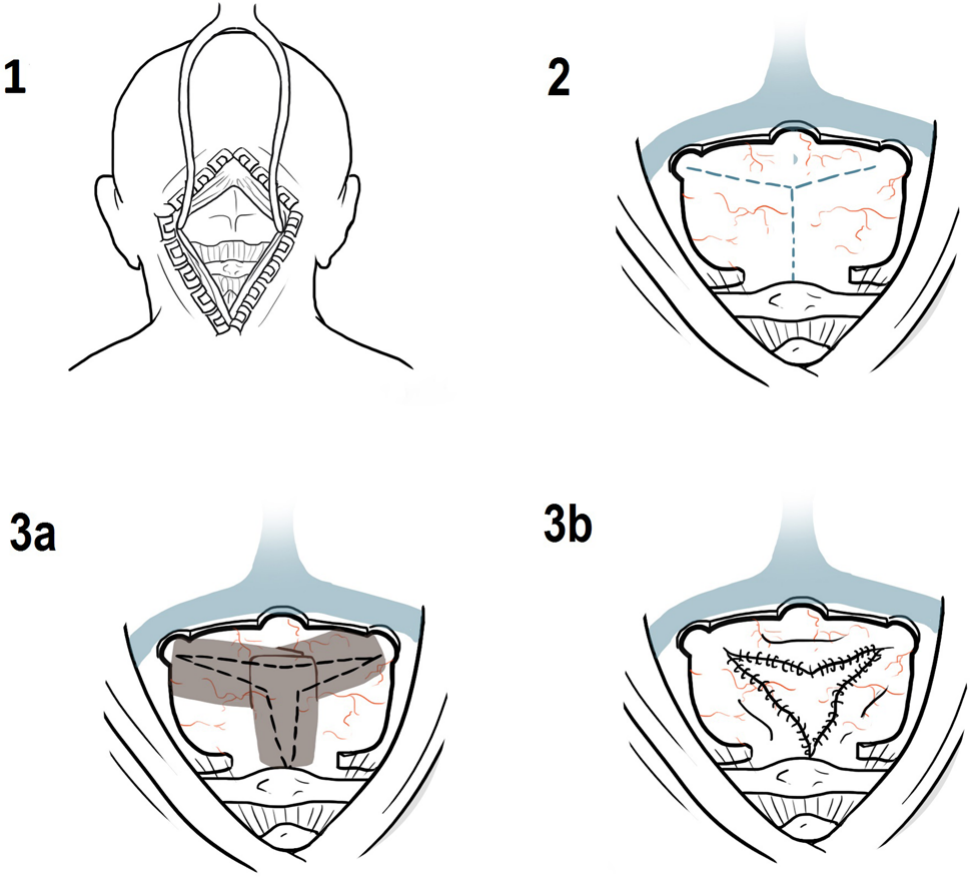
Suboccipital decompression. 1. Skin and muscle incision. 2. Bony decompression. 3a. Fibrin sealant patch closure (FSP group). 3b. Surgical reconstruction group with extension dural plastic (DR group)

We performed a single-center retrospective study of consecutive patients undergoing SDC for cerebellar hemorrhage, cerebellar infarction, and cerebellar subdural hematomas, treated between 01/2008 and 01/2020. Patients underwent computed tomography scan (CT) at admission, as well as CT-angiography scan (CT-A). We excluded patients with subarachnoid hemorrhage and hemorrhage from other vascular malformations, as well as benign or malignant oncological pathologies in the posterior fossa. Two surgical methods were performed depending on the officiating neurosurgeon: (1) fibrin sealant patch (FSP group), where fibrin sealant was used to cover the dural defect, without watertight suture or extension duraplasty, and (2) dural reconstruction group (DR) with artificial dural substitute or Musclepatch (Fig. 1). All patients received an external ventricular drain to prevent acute obstructive hydrocephalus. Clinical, radiological, and laboratory parameters (Quick, INR, aPTT, hemoglobin, and hematocrit) were included for further analysis. Furthermore, operative time, blood loss, and surgical technique as well as postoperative complications were analyzed. Finally, we performed the comparative analysis according to the underlying pathologies. The size of the mass lesions was measured in preoperative CT scan using ABC/2 method [18].

### Surgical technique

All decompressions were performed in standardized fashion. Patients were treated by external ventricular drainage (EVD) for hydrocephalus and SDC subsequently. The patient is placed in a prone position and the head is placed in the 3-pin Mayfield skullclamp maximum inclined. A 3-cm-wide strip is shaved along the planned incision. The skin and muscles are dissected and lifted of the bone (Fig. 1.1).

Bilateral SDC with extension to foramen magnum was performed in all patients. The margins of the SDC are placed as close as possible to sinus transversus and lateral sinuses [13]. The dura is opened widely in a stellate fashion (Fig. 1.2).

In the FSP group, the remaining dura is used to cover the brain tissue and Fibrin sealant patch is used just to cover the dural defect. No surgical duraplasty is performed, no patch or dural substitute is used (Fig. 1.3a).

In the DR group, an extension duraplasty is performed (Fig. 1.3b).

After the suboccipital craniectomy, the patients were evaluated for the necessity of permanent cerebrospinal fluid (CSF) drainage and received either a VP Shunt or the EVD was removed. Factors associated with the development of hydrocephalus, such as the length of the periprocedural external ventricular drain placement and chronic hydrocephalus development were examined. Functional neurological outcome was assessed with the modified Rankin scale (mRS) and stratified in favorable (mRS 0–2) vs. unfavorable (mRS 3–6).

### Statistics

Statistical analysis was performed using an unpaired *t*-test for parametric variables. Categorical variables were analyzed in contingency tables using the Fisher’s exact test. Results with *p* < 0.05 were considered significant. Data analyses were performed using the computer software package SPSS (version 25, IBM Corp., Armonk, NY). Mann–Whitney *U* test was used to compare the GCS by admission.

## Results

Between 01/2008 and 01/2020, SDC was performed in 338 patients. 251 patients were excluded due to: subarachnoid hemorrhage (*n* = 6), hemangioma (*n* = 29), tumor/metastasis (*n* = 154), Arnold-Chiari malformation (*n* = 38) and arteriovenous malformation (*n* = 24). Overall, 87 patients were included in the analysis (Table 1).

**Table 1:** Patient characteristics

Sex	
Male	50 (57.4%)
Female	37 (42.6%)
Underlying pathology	
Cerebellar infarction	29 (33.3%)
Cerebellar hemorrhage	56 (64.4%)
Suboccipital subdural hematoma	2 (2.3%)
Mean age (± SD) in years	66.3 ± 13.9
Number of Patients	87
FSP group	44 (50.5%)
SR group	43 (49.5%)

*SD* standard deviation, *FSP group* fibrin sealant patch group, *SR group* surgical reconstruction group

### Preoperative status

No significant differences in Quick, International normalized ratio (INR), activated partial thromboplastin time (aPTT), hemoglobin, platelet count, or platelet function testing (PFA) were found between the two groups. For detailed information, see Table 2.

**Table 2:** Preoperative coagulation state

	FSP group (*n* = 44)	DR group (*n* = 43)	
Platelet count	187 ± 67.4 G/l	196 ± 76.2 G/l	*p* = 0.56
Quick	83,3 ± 22.1%	83.3 ± 26.5%	*p* = 1.00
INR	1.2	1.2	
aPTT	24.6 s	25.7 s	
Hemoglobin	12.3 ± 1.9 g/dl	12.7 ± 2.4 g/dl	*p* = 0.39

*FSP group* fibrin sealant patch group, *DR group* dural reconstruction group, *INR* international normalized ratio, *aPTT* activated partial thromboplastin time

The median values of GCS between the groups were 6.0 in FSP group vs 7.5 in DR group, did not show any significant difference (*p* = 0.92).

### Intraoperative findings

Patients in the FSP group had significantly lower blood loss compared to patients in the DR group (341.4 ± 313.0 ml vs. 839.5 ± 508.2 ml; *p* < 0.0001).

Operative time was significantly shorter in the FSP group compared to the DR group (90.3 ± 31.0 min. vs. 199.0 ± 48.8 min; *p* < 0.0001). This statement is strongly limited by different factors: In four patients, second operation was necessary to acquire fascia lata, which was used as dural graft. Furthermore, the microscope was deemed necessary to perform the dural reconstruction, whereas in FSP group, dural closure was performed macroscopically. Finally, we found time-variations depending on the attending neurosurgical team.

### Postoperative complications

Overall, eight patients suffered from complications needing surgical revision. One patient in the FSP group underwent surgical revision for abscess formation and seven patients in the DR group underwent surgical revision (one patient for abscess, six patients for re-bleeding); *p* = 0.02.

### Hydrocephalus

There was no difference in the number of patients who developed hydrocephalus in both groups (6 in FSP group vs. 7 in DR group; *p* = 0.7). In the FSP group, five patients received VP-Shunt, whereas in the DR group, three patients received VP-Shunt. However, two patients in the FSP group and four patients in the DR group deceased because of non-neurosurgical complications before receiving a VP-Shunt. The mean length of external ventricular drainage placement was 8.7 ± 7.6 days for the FSP group vs. 8.4 ± 8.9 days for the DR group; *p* = 0.8.

### Outcome

16 patients in the FSP group vs. 13 patients in the DR group achieved favorable outcome at 6 months (*p* = 0.4).

### Underlying pathologies

We found no significant difference in distribution of underlying pathologies or size of the lesion among the groups.

We confirmed the significant difference in blood loss and operative time between the groups. For detailed information, see Table 2.

## Discussion

In patients undergoing SDC for cerebellar infarction or hemorrhage, various techniques exist to avoid CSF leakage, since this is one of the most common complications. On the other side, mass-effect lesions in the posterior fossa lead to direct compression of the brainsteam [15]. Patients suffering from such pathologies need rapid treatment [13] (Table 3). Decompression of the posterior fossa is established as an effective treatment. In many retrospective studies, various techniques are described from suboccipital craniotomy with removal of the hemorrhage or infarction, craniectomy without removal of the hemorrhage or infarction, or suboccipital craniectomy with removal of the hemorrhage or infarction [14]. Data regarding suboccipital decompression without watertight surgical reconstruction of dura mater is scarce. Previously, decompression without watertight surgical reconstruction of dura mater has already been introduced in supratentorial decompression [9, 10]. In the present study two techniques for dural reconstruction were performed according to the decision of the attending neurosurgeon, allowing a clear stratification of the patients.

**Table 3:** Underlying pathologies

	FSP ICH	DR ICH	
*n*	29	28	
Lesion size (cm^3^)	25.26 ± 15.6	26.92 ± 10.3	*p* = 0.63
OP-time (min)	91.17 ± 65.05	199.03 ± 68.23	*p* < 0.0001
Blood loss (ml)	362.0 ± 460.73	830.35 ± 532.60	*p* = 0.0008

	FSP CI	DR CI	
*n*	14	15	
Lesion size (cm^3^)	39.26 ± 14.5	36.14 ± 10.3	*p* = 0.50
OP-time (min)	89.64 ± 24.77	199.46 ± 58.46	*p* < 0.0001
Blood loss (ml)	346.42 ± 406.27	815.62 ± 524.92	*p* = 0.012

*FSP ICH* fibrin sealant patch group with intracerebellar hemorrhage, *FSP CI* fibrin sealant patch group with cerebellar infarction, *DR ICH* dural reconstruction group with intracerebellar hemorrhage, *DR CI* dural reconstruction group with cerebellar infarction, *OP-time* operative time, *N* number of patients

### Surgical complications

Patients in the DR group showed significantly higher rates of surgical revisions. There was no difference in infectious complications and wound healing disturbances. However, the bleeding complications were significantly more common among the patients in DR group (*p* = 0.01). Although the surgical technique had no influence on neurological outcome at 6 months in general, the neurological outcome of all patients with bleeding complications was unfavorable (90% deceased). A pathological preoperative coagulation state in any of the groups was ruled out.

### Hydrocephalus

The development of chronic hydrocephalus and the necessity for VP-Shunt placement did not differ between the groups. Mangubat et al. described a rate of CSF disturbances in patients with pathology in the posterior fossa at 15% and slightly higher incidence for ICH compared to cerebellar infarction (5/42, 11% ICH vs. 1/14, 7%) [11]. In our study, similar results were found (14%). We observed higher rates of permanent hydrocephalus in patients with cerebellar hemorrhage in comparison to cerebellar ischemia (9 vs. 4). In eight of nine patients with cerebellar hemorrhage, coexistent intraventricular hemorrhage was noted, which is a known predictor for hydrocephalus development [12, 14].

### Operative time

We confirmed the hypothesis of reducing time by using Fibrin sealant patch in suboccipital dura closure. The same has already been proven for supratentoriell decompressive craniectomy [9, 10]. Although being biased by several factors, the hypothesis of reducing time by using fibrin sealant patch in suboccipital dura closure was confirmed in our cohort*.* In general, shortening the duration of surgical procedures enhances their safety [16], especially when treating critically ill patients, and might reduce blood loss and rates of infection. It saves resources—in addition to the costs of the artificial implant—which is under the current health care policy of economic interest, as pointed out by Horaczek et al. [17]. As a result of the present retrospective institutional analysis, we changed our institutional treatment policy completely to dural closure via fibrin sealant patch alone due to shorter operation time, lower blood loss, reduced re-bleeding needing surgical revision, without influencing the rate of CSF leakage.

## Limitations

The present study has several limitations. Acquisition of data was retrospective and represents only a single-center experience. Furthermore, the type of dural closure technique was performed only according to the attending neurosurgeon. The use of external ventricular drain might limit the assessment of both dural reconstruction techniques. However, this might be partially outweighed by the fact that all patients received EVD before suboccipital decompression and was used for CSF drainage on a standardized protocol. The analysis of the operative time was strongly biased by different techniques of acquiring the patch (fascia lata vs. synthetical material) and the art of dural closure (microscopically in DR vs. macroscopically in FSP).

## Conclusion

Suboccipital decompressive craniectomy with Fibrin sealant patch dural closure is a safe procedure. Through this technique surgical time, blood loss, and complications needing surgical revision are reduced.

## Data Availability

Data available on request due to privacy/ethical restrictions.
